# Comprehensive analysis of blood cells and plasma identifies tissue-specific miRNAs as potential novel circulating biomarkers in cattle

**DOI:** 10.1186/s12864-018-4646-5

**Published:** 2018-04-10

**Authors:** Jason Ioannidis, F. Xavier Donadeu

**Affiliations:** The Roslin Institute and R(D)SVS, University of Edinburgh, Easter Bush, Midlothian, UK

**Keywords:** Circulating, microRNA, miRNA, Liver, Tissue-specific, Tissue-enriched, Biomarker, Cow, Small RNA sequencing, miR-802

## Abstract

**Background:**

The potential of circulating miRNAs as biomarkers of tissue function, both in health and disease, has been extensively demonstrated in humans. In addition, circulating miRNA biomarkers offer significant potential towards improving the productivity of livestock species, however, such potential has been hampered by the absence of information on the nature and source of circulating miRNA populations in these species. In addition, many miRNAs originally proposed as robust biomarkers of a particular tissue or disease in humans have been later shown not to be tissue specific and thus to actually have limited biomarker utility. In this study, we comprehensively analysed miRNA profiles in plasma and cell fractions of blood from cattle with the aim to identify tissue-derived miRNAs which may be useful as biomarkers of tissue function in this important food animal species.

**Results:**

Using small RNA sequencing, we identified 92 miRNAs with significantly higher expression in plasma compared to paired blood cell samples (*n* = 4 cows). Differences in miRNA levels between plasma and cell fractions were validated for eight out of 10 miRNAs using RT-qPCR (*n* = 10 cows). Among miRNAs found to be enriched in plasma, we confirmed miR-122 (liver), miR-133a (muscle) and miR-215 (intestine) to be tissue-enriched, as reported for other species. Profiling of additional miRNAs across different tissues identified the human homologue, miR-802, as highly enriched specifically in liver.

**Conclusions:**

These results provide novel information on the source of bovine circulating miRNAs and could significantly facilitate the identification of production-relevant tissue biomarkers in livestock. In particular, miR-802, a circulating miRNA not previously identified in cattle, can reportedly regulate insulin sensitivity and lipid metabolism, and thus could potentially provide a specific biomarker of liver function, a key parameter in the context of post-partum negative energy balance in dairy cows.

**Electronic supplementary material:**

The online version of this article (10.1186/s12864-018-4646-5) contains supplementary material, which is available to authorized users.

## Background

MicroRNAs (miRNAs) are short, non-coding RNA molecules which are primarily involved in the post-transcriptional fine-tuning of gene expression [1]. Since the discovery of lin-4 in *Caenorhabditis elegans* in 1993, thousands of miRNAs have been identified and characterised [2, 3]. It is now known that miRNAs are involved in the regulation of virtually all developmental processes, from the embryo to the adult [4–7], as well as in disease [8].

The discovery of cancer-associated miRNAs together with the detection of miRNAs in bio fluids [9] sparked a wave of research into the potential use of extracellular miRNAs as biomarkers of disease. A miRNA biomarker can be defined as a miRNA that is specifically produced or enriched in a given tissue, the circulating levels of which may reflect pathological or physiological changes in said tissue. In recent years, the value of circulating miRNAs as diagnostic biomarkers has been shown in relation to cancer (e.g. miR-21, miR-20, miR-221 [10, 11]), cardiovascular disease (miR-1, miR-133a [12]), liver disease (miR-122 [13]) and diabetes (miR-375 and miR-34 [14]), among many other human pathologies and physiological processes including pregnancy [15].

However, recent work has urged caution in interpreting the results of miRNA biomarker studies [16, 17]. Specifically, a large number of miRNAs initially proposed as cancer biomarkers were later shown not to be tissue-specific, contrary to what would be expected from a robust tissue biomarker. Many of those miRNAs were found to be present in blood cells, which are a major contributor of miRNAs in circulation [16], and their circulating levels were shown to be affected by changes in blood cell numbers. In addition, a review of non-cancer, disease-related miRNA biomarkers found that the majority of proposed biomarkers were not tissue-specific and, moreover, were not expressed in the disease-relevant tissues [18]. These reports highlight the need for further studies to identify the source of proposed miRNA biomarkers and determine their enrichment in the tissue(s) of interest.

The potential of miRNA biomarkers in food-producing species has also been reported, although in a much smaller number of studies than in humans. The identification of biomarkers associated with specific production traits and disease conditions in cattle would be of significant agricultural importance. A handful of studies have already linked circulating miRNA profiles with pregnancy and the oestrous cycle [19, 20], infection [21, 22] and grazing [23] in cattle, as well as with bacterial disease and feed deprivation in other domestic species [24]. However, the source and tissue-specificity of circulating miRNA populations in these species have not been determined which, in view of the above, limits the practical significance of those results.

To address these limitations and facilitate the discovery in cattle of circulating *bona-fide* miRNA biomarkers, we sought to profile miRNA populations in different blood fractions in order to distinguish miRNAs expressed in blood cells from those expressed in other body tissues which could provide novel specific biomarkers. Using next-generation sequencing, we provide a list of potential tissue-enriched miRNAs in circulation including miR-802, a previously uncharacterised bovine liver-specific miRNA which may prove useful as a disease biomarker in high-producing cows.

## Methods

### Sample collection

Blood samples were collected in EDTA-coated tubes (Becton Dickinson, USA) from the jugular vein of six non-pregnant Holstein-Friesian cross cows, aged between 20 and 25 months and held at Langhill Farm (University of Edinburgh), with the relevant ethics approval (see Declarations). From each blood sample, a plasma fraction was obtained using a two-step centrifugation protocol [19] to separate blood cells [25] followed by filtration through 0.2 μm syringe filters (Sartorius, Germany) to ensure the removal of any residual cellular debris. Subsequently, a cell fraction was obtained from a different volume (100 μL) of the same blood sample by centrifugation at 1900 x g for 10 min at 4 °C. The resulting supernatant was discarded and the cell pellet (consisting of red blood cell and buffy coat fractions) was re-suspended in RNase-free water up to a volume of 250 μL. The blood cell solution was immediately used for RNA extraction as described below.

Bovine tissue samples including brain, heart, intestine, kidney (cortex and medulla combined), liver, lung, skeletal muscle, ovary, placenta (placentome and interplacentome combined), skin, spleen and uterus were collected from a local abattoir in ice-cold PBS. Upon arrival in the laboratory tissues were dissected, labelled, weighed and snap-frozen in dry ice. All samples were stored at − 80 °C until further use.

### RNA extraction

All plasma, cell and tissue samples were extracted using TRIzol LS (Life Technologies, USA), as described [19], following the manufacturer’s protocol. Blood cells (250 μL) were homogenised using three volumes of TRIzol LS. Other tissues (each 50 mg) were thawed in 1 mL of TRIzol LS and homogenised with Lysing Matrix D (MP Biomedicals, UK) and a FastPrep FP120 Tissue Disruptor (Thermo Electron, USA). Before extraction, samples of plasma (700 μL) and blood cells (250 μL) were spiked with 3.5 μL of exogenous cel-miR-39-3p (5.6 × 10^8^ copies per sample, Qiagen), and glycogen (40 μg; Sigma-Aldrich, USA) was added in the presence of 1/10 per volume of 5 M ammonium acetate salt (Sigma-Aldrich), as recommended by the manufacturer, to facilitate RNA precipitation. RNA pellets from plasma, blood cells and other tissues were re-suspended, respectively, in 20, 10 and 40 μL of RNase-free water (Qiagen) and used immediately or frozen at − 80 °C until use. RNA content and quality from cell and tissue samples were determined using the Nanodrop ND-1000 Spectrophotometer (Thermo Fisher Scientific, USA).

### Small RNA sequencing

Small RNA libraries were prepared from four matched samples of blood plasma and cells using the TruSeq Small RNA Library Preparation Kit (Illumina, USA) and were submitted to 36-base single-end sequencing on the HiSeq 2000 Sequencing System (Illumina). Small RNA libraries were prepared using 5 μL of RNA extract. Raw sequencing data, available on the GEO database (Accession GSE84871 [26]), were analysed using sRNAbench 1.0 [27, 28]. Briefly, the software was run in genome mode using default settings, with the bovine genome (bosTau4) and miRBase 21 as reference (accessed on 30 April 2015 [29, 30]) in order to identify bovine (bta) miRNAs and human (hsa) miRNA homologues. Human homologues not previously described in bovine were identified by taking reads that aligned to the bovine genome but did not match with known bovine sequences in miRBase and aligning them to human sequences in miRBase. Throughout the manuscript, these miRNAs are indicated with the suffix hsa-. A single nucleotide mismatch was allowed when mapping reads to known miRNA sequences. Before mapping, reads without sequencing adaptor or with undetermined bases, and reads below 15 nucleotides in length (after adaptor removal) were removed and not used for subsequent analyses. For additional details about the integrated analysis steps in sRNAbench please refer to the software manual (http://bioinfo2.ugr.es:8080/ceUGR/srnabench/).

Normalised reads (reads per million mapped, RPMM) obtained from sRNAbench were filtered before being passed to edgeR 3.10.2 for differential expression analysis in R language 3.2.1 [31, 32]. Prior to analysis, miRNAs which were detected with less than 25 RPMM in more than two samples of either plasma or cells were excluded in order to increase confidence in subsequent analysis and reduce technical noise. Differential expression analysis was performed using edgeR’s paired mode on 212 miRNAs (Additional file 1) using GLMfit, as described in [33]. The statistical significance was set to false discovery rate (FDR) < 0.05.

### Identification of potential novel biomarkers in cattle

Potential novel tissue biomarkers were identified using the list of miRNAs which were enriched in plasma (Additional file 1). MiRNAs that were either registered for bovine but not human, or that were human homologues not previously identified in cow were selected. To do this, Ensembl genome browser 85 (www.ensembl.org, [34]), NCBI (www.ncbi.nlm.nih.gov) and miRBase 21 (www.mirbase.org [35]) were used to identify miRNAs annotated in cow and human, and to determine their conservation between the two species. BLAST was also used (accessed via Ensembl at www.ensembl.org/Multi/Tools/Blast?db=core, [36]) with default settings to identify the genomic location of selected miRNAs.

### RT-qPCR

MiRNA levels were quantified in matched blood plasma and cell samples. For plasma samples, 2 μL RNA were reverse-transcribed in a 10 μL reaction using the miScript II RT Kit (Qiagen) in a Whatman-Biometra Thermocycler (Biometra, USA). For blood cell and other tissue samples, 500 ng of RNA were used in each 10 μL reaction. The cDNA template was diluted 40-fold and added to 10 μL qPCR reactions which were prepared in 96-well format using the miScript SYBR Green PCR Kit (Qiagen). Template amplification was carried out in an Agilent MX3000P qPCR system (Agilent Technologies, USA). Raw fluorescence data were collected using MxPro software (Agilent Technologies). The amplification efficiency ranged between 84.5% and 117.4%, with R^2^ > 0.86. Expression levels were determined relative to a freshly-made standard curve and data were analysed using Microsoft Excel (Microsoft Corporation, USA). Expression levels were normalised to the mean expression of spiked-in cel-miR-39-3p for plasma and blood cells. For other tissues, expression data were normalised to RnU6–2.

Statistical analyses were carried out using GraphPad Prism 7 (GraphPad Software, USA). Differences in miRNA expression between blood plasma and cell samples were assessed using paired t-tests. In all cases, normality was tested using the Shapiro-Wilk normality test, outliers were tested with the ROUT test, and data were log_2_(x + 1) transformed to meet the tests’ normality criteria. Statistical significance was set to *P* < 0.05.

## Results and discussion

### Small RNA sequencing of different blood fractions

**Fig.1:**
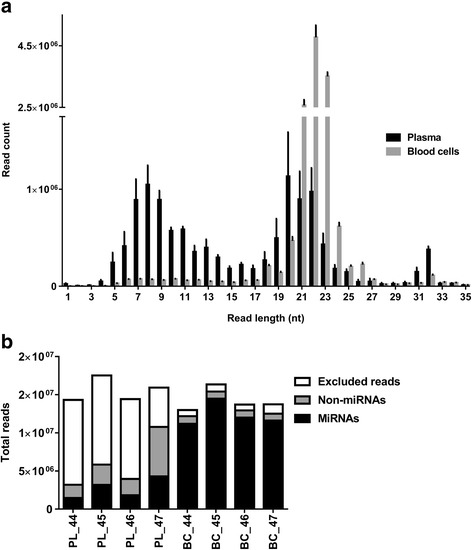
Results of small RNA sequencing analysis. **a** Length distribution (mean ± SEM) of reads (before filtering out short reads) from paired plasma and cell samples of blood from four animals. **b** Bar plot showing the relative distribution of sequencing reads corresponding to miRNAs, non-miRNAs and excluded reads in individual blood plasma (PL) and cell (BC) samples

To determine the relative abundance of miRNAs in different blood compartments, we sequenced miRNAs in paired blood plasma and cell fractions from four cows. Analysis of read length distributions showed a peak at 20–23 nucleotides (characteristic of mature miRNAs) in blood cells and, although of lesser magnitude, in plasma (Fig. .1a). Unlike blood cells, plasma samples displayed an additional peak at 7–9 nucleotides. We attribute the higher proportion of short RNA fragments in plasma relative to cell samples to 1) the higher RNase content in plasma [9] resulting in higher levels of RNA degradation and 2) the relatively lower abundance of RNAs in plasma resulting in libraries with a higher proportion of adapter/adapter complexes. The difficulty associated with visualising low abundance cDNA bands on a gel during preparation of the plasma library likely resulted in many of the shorter sequences being included for sequencing.

Averaged for both plasma and cells, we obtained 14.9 million raw reads per sample (Table 1). After removing reads with low quality (reads without adapter, short reads and reads with multiple undetermined base calls), we mapped on average 5.9 million and 13.3 million reads to the bovine genome (see Methods) which correspond to 38.1% and 94.3% of the total reads for plasma and cells, respectively (Table 1, Fig. .1b). On average, plasma generated 2.7 million miRNA reads compared to 12.3 million reads for cells, the majority of which were, in both cases, bovine miRNAs (Table 1, Fig. .1b). After applying a detection threshold of 10 reads per sample, we detected up to 315 unique miRNAs in plasma samples and 305 in cell samples, of which only 17 and 16, respectively, were human homologues (Table 1).

**Table 1:** Distribution of sequencing reads (RPMM) from paired blood plasma and cell samples (*n* = 4 cows)

	Plasma	Cells
Raw reads	15,553,150	14,194,750
Reads without adapter (excluded)	127,738	237,726
Short reads (excluded)	9,469,537	672,891
Low quality reads (excluded)	20,587	25,532
Mapped reads	5,935,288	13,258,602
Total miRNA reads	2,688,243	12,309,110
of which bovine	2,683,651	12,307,733
of which human	4592	1377
Detected miRNAs (> 10 reads)	315	305
of which bovine	298	289
of which human	17	16

### Differentially expressed miRNAs in blood plasma and cells

There was incomplete overlap between the lists of most abundant miRNAs in plasma and cells, with only 5 of the 15 most abundant miRNAs being common to the two lists, i.e. miR-486, miR-142-5p, miR-191, miR-92a and miR-30e-5p (Table 2).

**Table 2:** Most abundant miRNAs in paired blood plasma and cell samples (*n* = 4 cows) obtained by sequencing

Plasma		Cells	
MiRNA	RPMM	MiRNA	RPMM
miR-22-3p	126,167	miR-486	324,749
miR-486	125,775	miR-451	187,081
miR-192	101,853	miR-101	90,473
miR-27b	43,371	miR-92a	44,722
miR-142-5p	39,727	miR-16b	33,980
miR-423-5p	35,409	miR-26a	32,849
miR-191	32,448	miR-25	30,232
miR-215	28,947	miR-142-5p	20,925
miR-21-5p	25,484	miR-191	19,656
miR-10b	25,277	miR-181a	15,753
miR-92a	23,608	miR-186	14,137
miR-103	22,481	let-7f	12,830
miR-140	19,501	miR-30e-5p	11,955
miR-148a	19,108	miR-144	9847
miR-30e-5p	19,058	miR-93	9760

**Fig. 2:**
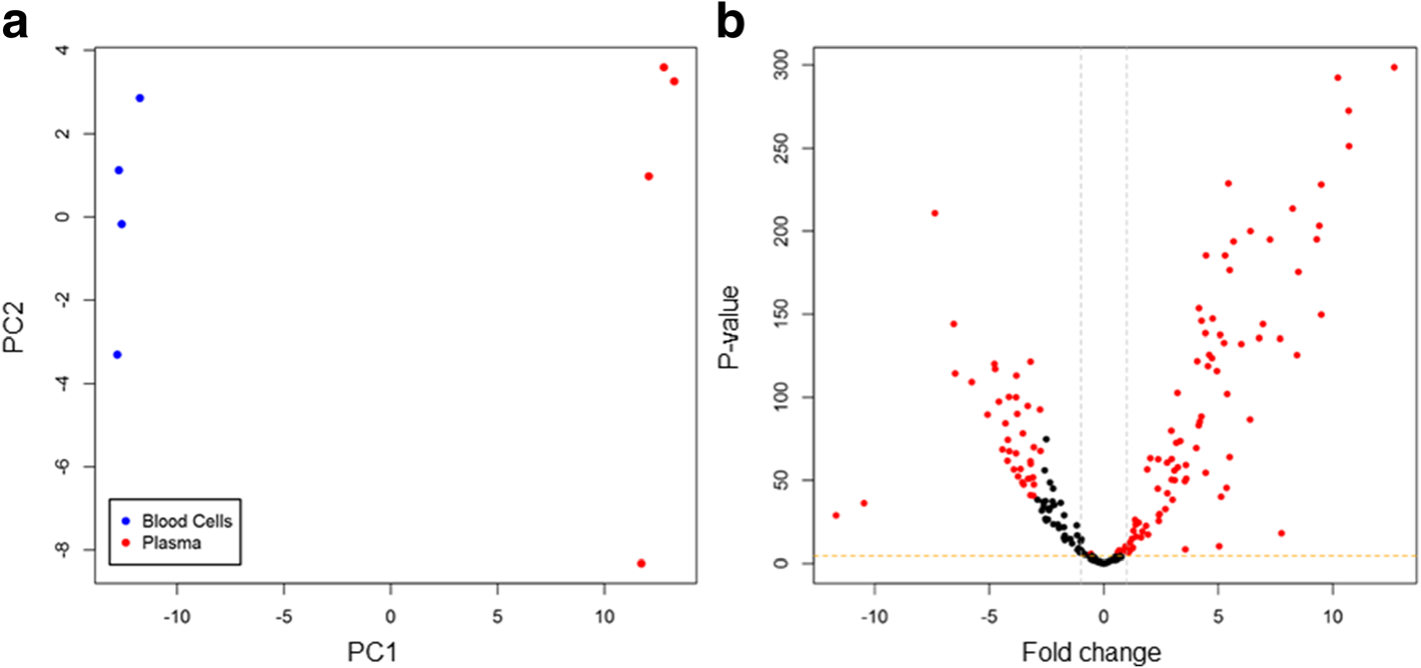
Small-RNA sequencing results. **a** PCA plot showing the first two principle components using transformed normalised data. **b** Volcano plot of fold-change (miRNA levels in blood plasma relative to cells) versus statistical significance (*P*-values), using transformed normalised data. MiRNAs with fold-change > 2 and FDR < 0.05 are highlighted in red. Grey dotted lines indicate two-fold difference thresholds. The orange dotted line indicates *P* = 0.05. Paired blood plasma and cell samples from four animals were analysed

**Fig. 3:**
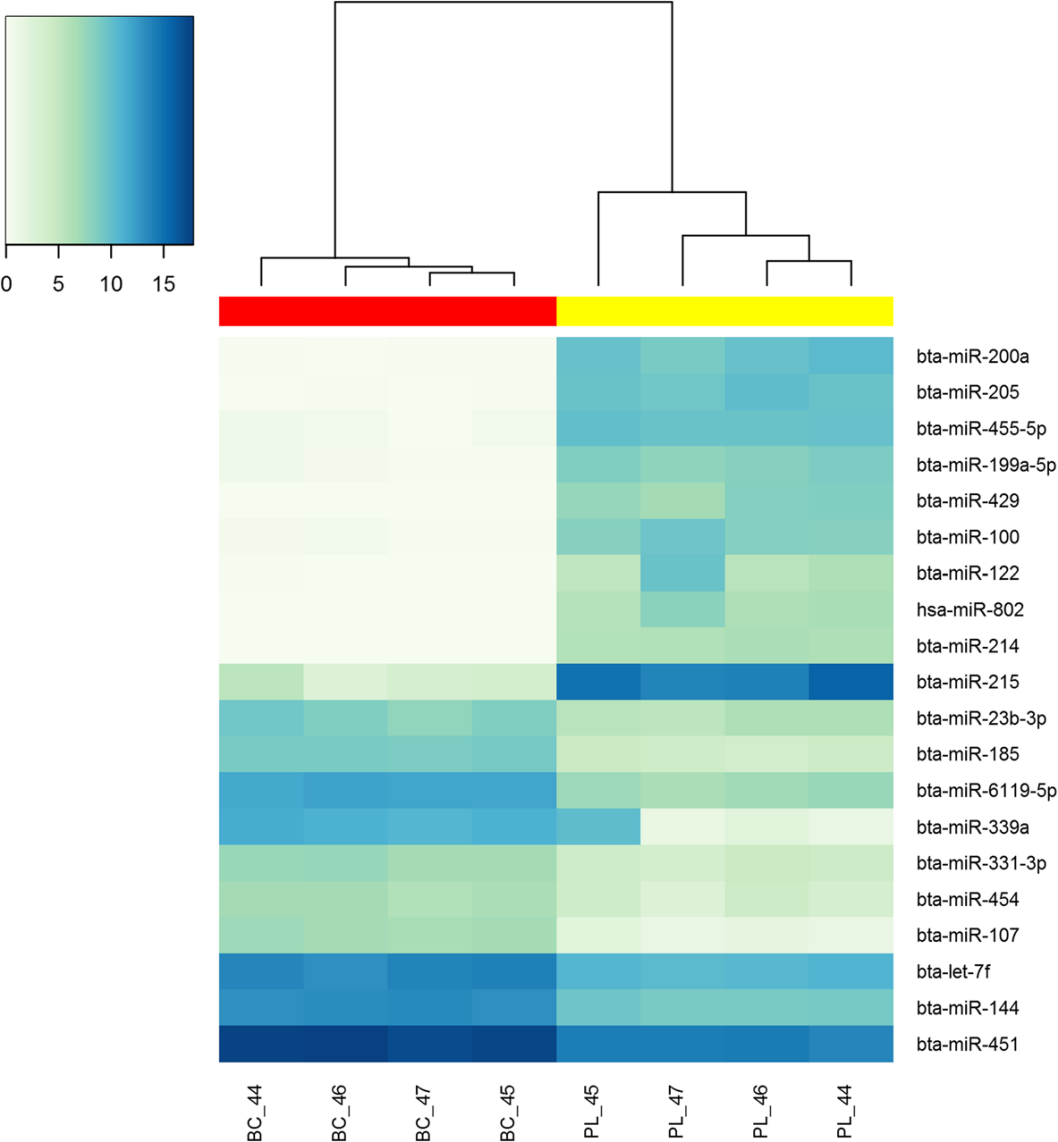
Heat map of top miRNAs differentially expressed in paired samples of blood plasma (PL) and cells (BC). Each row in the heat map represents a miRNA and each column represents a sample. The colour scale illustrates the relative expression level of miRNAs. FDR < 0.05, *n* = 4 cows

Principal component analysis (PCA) of a total of 212 miRNAs that were detected with ≥25 RPMM in more than two samples of either plasma or cells (see Methods, Additional file 1) produced well-separated plasma and cell populations, with a single component (PC1) accounting for 83.9% of the variation across samples (Fig. 2a). Moreover, 169 miRNAs were differentially expressed between plasma and cells (FDR < 0.05, Fig. 2b, Additional file 1), with as many as 131 miRNAs changing by more than two-fold. Of these, 92 miRNAs were enriched in plasma and 39 miRNAs were enriched in blood cells (Table 3, Fig. 3 and Additional file 1). Among miRNAs enriched in plasma were several known to be expressed exclusively or predominantly in specific tissues in humans including miR-122 (liver), miR-133a (muscle), miR-127 (adrenal gland), miR-141 (adrenal gland and reproductive system) and miR-182 (thymus) [37, 38]. Moreover, miRNAs enriched in the bovine blood cell fraction included many previously reported to be blood cell-derived in humans, such as miR-144, miR-451, let-7f, miR-26a, miR-15b, miR-20a, miR-16a, miR-16b and miR-486 [18, 25, 37, 39–41].

**Table 3:** The 15 most enriched miRNAs in blood plasma and cell samples (n = 4 cows) obtained by sequencing

Plasma-enriched miRNAs	Fold-change Plasma/Cells	Blood cell enriched miRNAs	Fold-change Cells/Plasma
miR-429	Expressed exclusively in plasma	miR-107	54.19
miR-200a	6503.71	miR-6119-5p	28.01
miR-205	5220.54	miR-185	27.94
miR-215	2168.64	miR-144	16.56
miR-455-5p	1707.35	miR-451	10.31
miR-100	1498.54	miR-331-3p	8.55
miR-199a-5p	1359.19	miR-454	7.92
miR-10b	1339.56	miR-339a	7.61
hsa-miR-802	1092.70	let-7f	7.16
miR-199c	1041.52	miR-23b-3p	6.53
miR-214	931.10	miR-26a	6.08
miR-141	699.78	miR-98	5.72
miR-455-3p	635.12	let-7a-5p	5.60
miR-122	493.42	miR-101	5.39
miR-126-5p	412.95	miR-197	5.36

FDR < 0.05 for all miRNAs

### Validation of sequencing results using RT-qPCR

**Fig. 4:**
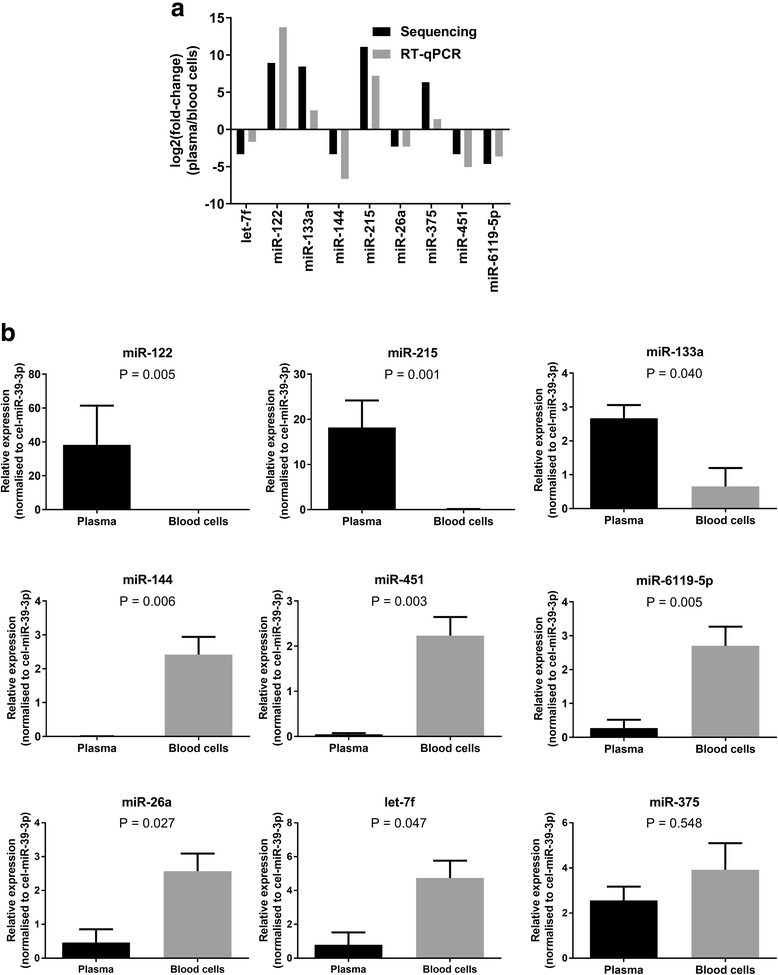
Validation of sequencing results. **a** Comparison of the fold-changes in miRNA levels in blood plasma relative to cells obtained using sequencing and RT-qPCR. **b** Relative expression (mean ± SEM, calculated from a standard curve) of selected miRNAs in paired blood plasma and cell samples from six cows. All expression levels were normalised to cel-miR-39-3p

We used RT-qPCR to validate differences involving a total of 10 miRNAs (indicated in bold in Additional file 1) using samples from a total of six animals (including the four animals used for sequencing). Levels of one of the miRNAs selected for validation (miR-429) could not be accurately quantified in most samples because of its low abundance. For the remaining nine miRNAs (Fig. 4a) we confirmed differences (*P* < 0.05) in the levels of all but miR-375 between plasma and cell samples (Fig. 4b). We then sought to validate the results for these eight miRNAs (miR-122, miR-215, miR-133a, miR-144, miR-451, and miR-6119-5p, miR-26a and let-7f) using samples from an independent group of animals (four Holstein-Friesian cross cows, aged between 24 and 48 months, in late pregnancy or post-partum) and we confirmed differences in plasma and cell levels of seven miRNAs (Additional file 2); levels of miR-133a were very low (Ct 34–37) in the original group of animals and were undetectable in this second group.

### MiRNA profiling across bovine tissues

**Fig. 5:**
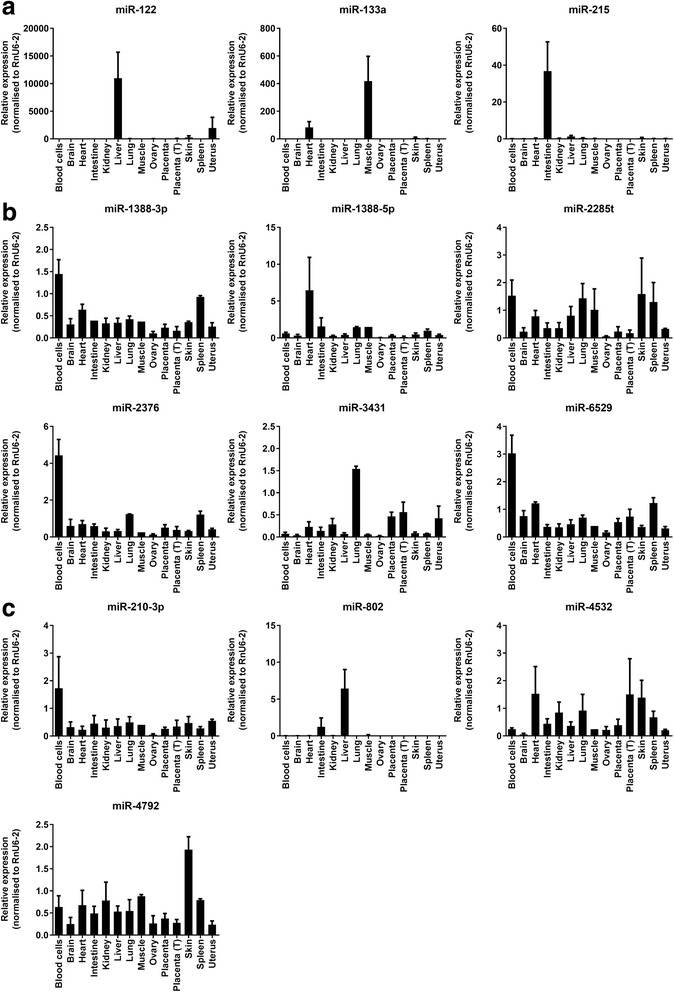
MiRNA profiling across bovine tissues. Relative expression (mean ± SEM) of selected plasma-enriched miRNAs across 14 bovine tissues. **a** MiRNAs reported to be tissue-enriched in humans. **b** MiRNAs not registered in humans. **c** Human miRNA homologues not identified previously in bovine. RT-qPCR was run on 2–6 samples per tissue. Placenta = Day 70 of pregnancy; Placenta (T) = Term. All expression levels were normalised to RNU6–2

To identify novel tissue-specific miRNAs in cattle we investigated the distribution across 14 different body tissues of several miRNAs which expression we had found to be enriched in plasma (Additional file 1). We first profiled three miRNAs, miR-122, miR-133a and miR-215, known to be tissue-specific in humans [37, 38]. Accordingly, the three miRNAs (Fig. 5a) were expressed predominantly in liver (miR-122, 88-fold higher than in any other tissue), muscle/heart (miR-133, 254-fold higher) and intestine (miR-215, 150-fold higher).

We then sought to identify novel tissue biomarkers. First, from the list of miRNAs enriched in plasma (Additional file 1), we looked for miRNAs that were either registered for bovine but not human, or that were human homologues not previously identified in cow, as described in the Materials and Methods. These analyses revealed 10 miRNAs which either were present in bovine but not in human (miR-1388-5p, miR-1388-3p, miR-2376, miR-2285 t, miR-3431 and miR-6529a, Fig. 5b) or were homologues of human miRNAs not identified previously in cow (miR-210-3p, miR-802, miR-4532 and miR-4792, Fig. 5c).

RT-qPCR analyses showed that the majority of these miRNAs were expressed in most of the tissues profiled, i.e., they were not enriched in any particular tissue and therefore do not provide potential biomarkers of tissue function (Fig. 5b-c). Surprisingly, miR-1388-3p, miR-2376 and miR-6529 showed high expression in blood cells despite having been identified as plasma-enriched using sequencing. A possible explanation for this result is that, despite blood cells having the highest levels of these miRNAs, the combined contribution from all other tissues to plasma levels is still greater in comparison. Interestingly, in addition to blood cells, all three miRNAs were relatively abundant in spleen (Fig. 5b), which suggests an involvement in hematopoietic regulation. However, since no functional data is available for any of the three miRNAs in any species, further study will be needed to confirm this.

Among the remaining miRNAs, miR-3431 was distinctly high in lung followed by placenta and uterus. This is a ruminant-specific miRNA which has been reported to be expressed in adipose and reproductive tissues of cattle [42, 43], although no information is available on its potential functions. Whether miR-3431 could provide a useful circulating biomarker of lung function in cows should be investigated in future studies.

Of note, the human homologue of miR-802 was distinctly enriched in liver, where its expression was 87-fold higher than the mean expression across all tissues, and 5.2-fold higher than in intestine, the tissue with the second highest mean expression levels (Fig. 5c). Using BLAT (UMD3.1, accessed 27/07/16) we mapped the mature miR-802 sequence to bovine chromosome 1 (chr1: 149720302–149,720,392 [+]). Indeed, this location in the bovine genome has been annotated as a novel miRNA with a secondary structure that matches miR-802. Although not previously reported in cow, data in humans and mice have shown miR-802 to be a key a regulator of liver function. Specifically, hepatic levels of miR-802 were elevated in obese individuals, contributing to insulin insensitivity, glucose intolerance and increased hepatic gluconeogenesis by downregulation of HNF1B [44]. Interestingly, serum miR-802 levels were reportedly increased in type 2 diabetes patients providing a potential circulating biomarker for this disease [45]. Another study identified miR-802 as a target of constitutive androstane receptor (CAR) signalling, which is involved in the regulation of xenobiotic detoxification, lipid homeostasis and energy metabolism [46]. Furthermore, circulating miR-802 has been proposed as a biomarker of drug-induced liver damage in rats, with an observed increase in miRNA levels in response to tissue damage being comparable to that of the liver-enriched miR-122 [47, 48]. Overall, these results highlight the importance of miR-802 in the regulation of glucose and lipid metabolism as well as xenobiotic responses in the liver.

In dairy cattle, negative energy balance (NEB) often occurs during the post-partum period as a consequence of the high energy requirements for lactation. The liver has a central role in counteracting NEB. Liver dysfunction often ensues during the post-partum period, particularly in high-producing cows, in association with metabolic dysfunction and low fertility, constituting a major problem in modern dairy herds [49]. Changes in the expression of some miRNAs in liver have been shown in association with NEB in cattle [49]. In this context, future studies should investigate whether miR-802, perhaps in combination with liver-enriched miR-122, may provide a potential biomarker of negative energy balance in dairy cows.

## Conclusions

Using next-generation sequencing, we compared for the first time miRNA levels in paired plasma and cell fractions of bovine blood and identified a total of 131 miRNAs that were differentially expressed (greater than two-fold) between the two fractions. Of those, 92 miRNAs were expressed at higher levels in plasma than in cells. Expression profiling of selected plasma-enriched miRNAs across different bovine tissues revealed miR-802, a previously unidentified bovine miRNA, to be highly enriched in liver. The miRNA database generated in this study provides a useful resource for future investigation of miRNA biology in bovine and identification of additional potential biomarkers of tissue function in livestock.

## Additional files

Additional file 1: Sequencing miRNA analysis. Normalised miRNA expression levels in bovine blood plasma and cell samples, and the results of differential expression analysis between the two sample types. (XLSX 47 kb)

Additional file 2: Validation of sequencing data. Validation of sequencing results for selected miRNAs using RT-qPCR on an independent group of four animals. (TIFF 1669 kb)

## Abbreviations

miRNA: microRNA
PCA: Principal component analysis
qPCR: Quantitative polymerase chain reaction
RT: Reverse transcription
