# Supplementary figures and images for: Comprehensive analysis of blood cells and plasma identifies tissue-specific miRNAs as potential novel circulating biomarkers in cattle
Source: BMC Genomics. 2018 Apr 10;19:243. doi: 10.1186/s12864-018-4646-5 (PMC5894187; doi:10.1186/s12864-018-4646-5)

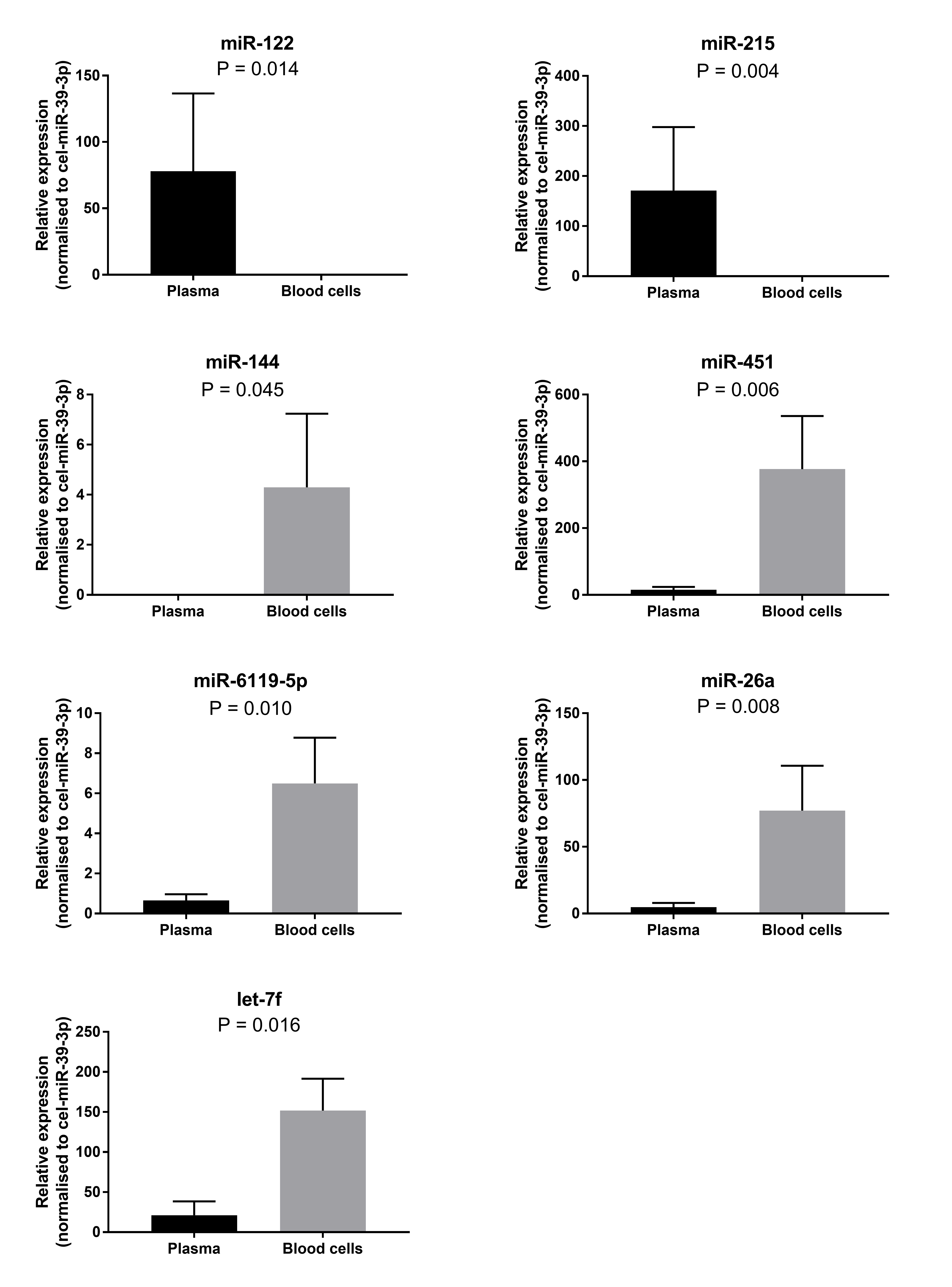

Supplement: Supplementary file 2 — Validation of sequencing data. Validation of sequencing results for selected miRNAs using RT-qPCR on an independent group of four animals. (TIFF 1669 kb) [file 12864_2018_4646_MOESM2_ESM.tif]
